# The *Arabidopsis* PLAT Domain Protein1 Is Critically Involved in Abiotic Stress Tolerance

**DOI:** 10.1371/journal.pone.0112946

**Published:** 2014-11-14

**Authors:** Tae Kyung Hyun, Eric van der Graaff, Alfonso Albacete, Seung Hee Eom, Dominik K. Großkinsky, Hannah Böhm, Ursula Janschek, Yeonggil Rim, Walid Wahid Ali, Soo Young Kim, Thomas Roitsch

**Affiliations:** 1 Institute of Plant Sciences, University of Graz, Graz, Austria; 2 Plant Molecular Biology and Biotechnology Research Center, Gyeongsang National University, Jinju, Korea; 3 Department of Plant and Environmental Sciences, Copenhagen Plant Science Centre, University of Copenhagen, Taastrup, Denmark; 4 Departamento de Nutrición Vegetal, CEBAS-CSIC, Campus Espinardo, Murcia, Spain; 5 Department of Pharmaceutical Biology, University of Würzburg, Würzburg, Germany; 6 Department of Molecular Biotechnology and Kumho Life Science Laboratory, College of Agriculture and Life Sciences, Chonnam National University, Gwangju, Korea; 7 Global Change Research Centre, CzechGlobe AS CR, v.v.i., Drásov, Czech Republic; University of South Florida College of Medicine, United States of America

## Abstract

Despite the completion of the *Arabidopsis* genome sequence, for only a relatively low percentage of the encoded proteins experimental evidence concerning their function is available. Plant proteins that harbour a single PLAT (**P**olycystin, **L**ipoxygenase, **A**lpha-toxin and **T**riacylglycerol lipase) domain and belong to the PLAT-plant-stress protein family are ubiquitously present in monocot and dicots. However, the function of PLAT-plant-stress proteins is still poorly understood. Therefore, we have assessed the function of the uncharacterised *Arabidopsis* PLAT-plant-stress family members through a combination of functional genetic and physiological approaches. *PLAT1* overexpression conferred increased abiotic stress tolerance, including cold, drought and salt stress, while loss-of-function resulted in opposite effects on abiotic stress tolerance. Strikingly, PLAT1 promoted growth under non-stressed conditions. Abiotic stress treatments induced *PLAT1* expression and caused expansion of its expression domain. The ABF/ABRE transcription factors, which are positive mediators of abscisic acid signalling, activate *PLAT1* promoter activity in transactivation assays and directly bind to the ABRE elements located in this promoter in electrophoretic mobility shift assays. This suggests that *PLAT1* represents a novel downstream target of the abscisic acid signalling pathway. Thus, we showed that PLAT1 critically functions as positive regulator of abiotic stress tolerance, but also is involved in regulating plant growth, and thereby assigned a function to this previously uncharacterised PLAT domain protein. The functional data obtained for PLAT1 support that PLAT-plant-stress proteins in general could be promising targets for improving abiotic stress tolerance without yield penalty.

## Introduction

The PLAT domain (PS50095; **P**olycystin-1, **L**ipoxygenase, **A**lpha-toxin and **T**riacylglycerol lipase) forms a β-sandwich composed of two sheets of four strands each and is an intracellular domain. It occurs in a variety of membrane or lipid associated proteins that are multi-domain proteins, but also in proteins harbouring either a single PLAT domain or repeats [1]–[5]. Because of its similarity to the C2 domain, the PLAT domain was proposed to function in protein-protein interactions as well as protein-membrane interactions [2], [4], [6]. Indeed, the PLAT domain of *Caenorhabditis elegans* polycystin LOV-1 and human polycystin-1 interact with ATP-2, an ATP synthase F1 subunit [7], while the human polycystin-1L2 interacts with different types of G-proteins [8]. Importantly, the association with membranes is essential for the proper function of PLAT domain proteins [2], [7]. Further, the membrane targeted 11*R*-Lipoxygenase from *Gersemia fruticosa* was shown to bind calcium, required to induce its activity [9]. The PLAT domain regulates the catalytic activity in multi-domain proteins, but also in proteins interacting with the PLAT domain [2], [6], and was shown to regulate substrate specificity [10]. Whereas substantial experimental data on PLAT domain proteins is available for the animal field, PLAT domain proteins from plants were only poorly studied, despite the fact that genes encoding PLAT domain proteins were isolated from several plant species [3], [4], [11]–[16].

Transgenic approaches to improve abiotic stress tolerance often resulted in yield penalties under optimal growth conditions [17], [18], while only few studies reported an associated improved plant growth [4], [19]. Interestingly, one of these studies addressed the so far only studied PLAT-plant-stress protein *Ca*TIN1 from *Capsicum annuum*, however this protein was only studied using heterologous expression in tobacco [4]. Because both gain-of-function and antisense *CaTIN1* expression promoted abiotic and biotic stress tolerance, *Ca*TIN1 function remained elusive. Proteins that belong to the PLAT-plant-stress protein family (Conserved Domain cd1754) are ubiquitously present in monocot and dicot plant species and harbour a single PLAT domain. Our analyses of the limited *in silico* expression data available for PLAT-plant-stress proteins indicate transcriptional induction by different abiotic and biotic stimuli. This suggests that PLAT-plant-stress proteins in general could promote tolerance towards stress responses, although no data from functional studies in homologous systems are available for these proteins.

The plant hormone abscisic acid (ABA) regulates different aspects of plant development, such as stomatal aperture [20] and seed germination [21]. ABA production is increased by abiotic stresses and ABA regulated genes strongly overlap with those induced under drought, salinity and less prominently cold stress conditions [22]–[25]. The ABA stimulated stomatal closure has been shown to serve as primary defence mechanism during the initial phase of biotic stress responses [26], [27]. In contrast, ABA mostly negatively regulates the subsequent phases in biotic stress responses by repressing the salicylic acid, ethylene, jasmonic acid and cytokinin signalling pathways [26]–[29]. ABA-deficient mutants showed enhanced defence responses against *Botrytis cinerea* [30], and virulent bacteria in tomato [31] and *Arabidopsis* [32]. Although these findings suggest that ABA is involved in the crosstalk between abiotic and biotic stress responses, no direct link in the antagonistic interaction between these stresses is available.

Based on comparative genomic analysis, we identified three *Arabidopsis* genes (*PLAT1 AT4G39730*, *PLAT2 AT2G22170* and *PLAT3 AT5G65158*) that belong to the PLAT-plant-stress subgroup and submitted this annotation to the TAIR database. PLAT1 and PLAT2 are orthologs of *Ca*TIN1 and *Ca*TIN1-2, respectively [4], [16]. Our *in silico* analysis of published experimental data [33] revealed that cold stress induced the expression levels for the *PLAT1* ortholog in *Thlaspi arvense*, which is a close relative of *Arabidopsis*. Based on these findings we hypothesised that the *Arabidopsis* members from this PLAT-plant-stress subgroup, similar to *Ca*TIN1 [4], also promote tolerance towards various stress responses. Here we report on the molecular characterisation and functional analysis of the PLAT-plant-stress subgroup family member AT4G39730 that we designated as *Arabidopsis* PLAT domain protein 1 (PLAT1). We showed that *PLAT1* critically functions as positive regulator of abiotic stress tolerance, also promotes plant growth and is a direct target of the ABF transcription factors, which are positive mediators of the ABA signalling pathway [34], [35]. The possible practical application to increase abiotic stress tolerance without yield penalty in crop species is discussed.

## Materials and Methods

### Plasmid construction and plant transformation

Total RNA isolated from *Arabidopsis* (TRIR reagent from Thermo Fischer Scientific, Germany) was reverse-transcribed using the ReverAid™ First strand cDNA synthesis kit (Thermo Fischer Scientific, Germany). Using this cDNA as template, the full-length *PLAT1* cDNA was amplified by PCR with the PLAT1-F cDNA and PLAT1-R cDNA primers (Table S1 in File S1). The dexamethasone inducible overexpression construct, *35S*>>*PLAT1* was created by cloning the *PLAT1* cDNA PCR product in the *OP* shuttle vector *pEG647*. The resulting *OP:PLAT1* cassette was transferred to the binary vector *pEG618* harbouring the *35S:LhGR* activator component, resulting in *35S*>>*PLAT1*. The *PLAT1* rescue/reporter constructs were generated by PCR amplification from genomic DNA with the PLAT1-F and PLAT1-R rescue primers (Table S1 in File S1) to isolate the genomic fragment harbouring 2039 bp *PLAT1* promoter sequence, including the 5′-UTR region, and the *PLAT1* coding region without stop codon. Subsequently, the venus YFP or GUS reporter proteins were fused in frame to the C-terminus of the PLAT1 protein resulting in *PLAT1:PLAT1-YFP* and *PLAT1:PLAT1-GUS*, respectively. The different *35S:ABF_1–4_* overexpression constructs were created by cloning the *ABF_1–4_* PCR products (ABF_1–4_-F OX and ABF_1–4_-R OX primers, Table S1 in File S1) into the *pPS1* binary vector. The MBP-ABF_1–4_ fusion proteins for the EMSA experiments were created by cloning the ABF_1–4_ PCR products (ABF_1–4_-F and ABF_1–4_-R primers; Table S1 in File S1) into the *pMA-c2xL* vector harbouring the maltose binding protein as tag for protein purification. Binary vectors were introduced into *Agrobacterium tumefaciens* LBA4404 by electroporation and used to transform *Arabidopsis* plants using the floral dip method [36].

### Phylogenetic analysis

To identify members of the PLAT-plant-stress family from other plant species, multiple database searches were performed using the Basic Local Alignment Search Tool (BLAST) algorithms BLASTp and tBLASTn available on the public databases, PLAZA 2.0 (bioinformatics.psb.ugent.be/plaza) and Phytozome v8.0 (www.phytozome.net) with cutoff value of E<10^−5^. We used nucleotide and amino acid sequences of PLAT1 from the TAIR database (www.arabidopsis.org) to BLAST all databases. Phylogenetic analysis was performed by using CLUSTALW alignment in PHYLIP format clustal algorithm, and displayed in a phylogram tree format with locus name of each protein. Bootstrap values were presented as a percent of 100 resampled trees at each tree node using default settings of the TreeTop-Phylogenetic Tree (www.genebee.msu.su/services/phtreereduced.html).

### Plant materials and growth conditions

*Nicotiana benthamiana* plants were grown under greenhouse conditions as described previously [37]. *Arabidopsis* plants (Col-0 ecotype) were grown in soil at 8 h light/16 h darkness at 22°C (light intensity: 180 µmol m^−2^ s^−1^) or on half strength MS medium under continuous light at 22°C (light intensity: 180 µmol m^−2^ s^−1^) in growth cabinets. T3 homozygous T-DNA insertion lines were obtained for the *PLAT1* gene, *plat1-1* SALK-112728c and *plat1-2* SALK-1283454c, and the *PLAT2* gene, *plat2* SAIL-1171C06. The T-DNA insertions were verified with the primers PLAT1-1-F, PLAT1-1-R and SALK LB2 (*plat1-1*), PLAT1-2-F, PLAT1-1-R and SALK LB2 (*plat1-2*), and PLAT2-F, PLAT2-R and pROK2 LB1 (*plat2*) (Table S1 in File S1). For all plant experiments T3 or T4 homozygous plant lines were employed, based on the segregation of the respective antibiotic selection marker, except for the experiments shown in Figure S4, for which segregating T2 *Arabidopsis* lines were employed.

### Abiotic stress conditions

The abiotic stress experiments in soil were performed as 3 biological replicates (cold stress as 2 biological replicates) with at least 10 plants each. For the *Arabidopsis* germination experiment, seeds were directly germinated on half strength MS medium including the respective chemicals as indicated. At least 100 seeds per treatment/genotype were used in 3 independent experiments. For the salt stress tolerance in plates, seeds were germinated and grown for 6 d on half strength MS medium, transferred to half strength MS medium including NaCl (either 0, 150 or 200 mM) and optionally 5 µM dexamethasone (*35S>>PLAT1* lines), and grown for another 4 d. These experiments were performed as 2 biological replicates, each with 2 technical replicates and with >12 Col-0 and >26 mutant/transgenics seedlings per plate. For the tunicamycin (TM) experiments, seeds were directly germinated on half strength MS medium including different TM concentrations. At least 70 seeds per treatment/genotype were used in 3 independent experiments, with 2 technical replicates each.

### Biotic stress conditions

To determine pathogen susceptibility, leaves from 8-w-old *Arabidopsis* plants were infected with *Pseudomonas syringae* pv. *tomato* DC3000 with or without the *avrRpm1* gene by infiltration using a needleless syringe as described previously [38]. Visual evaluation of disease symptoms were conducted at 3 to 5 d. For expression analysis, 8-w-old plants were infected with *P. syringae* pv. *tomato* DC3000 or *Sclerotinia sclerotiorum*. Subsequently, the *S. sclerotiorum* infected plants were kept in a clear plastic box under saturating humidity. The biotic stress experiments were performed as 3 biological replicates with at least 10 plants each.

### Expression analysis

Total RNA isolation and Northern-blot analysis was carried out as described previously [37]. Filters were exposed to a screen for 4 d, which was scanned with a Phosphor imager (Fuji BAS2000, Ray-test, Germany). The probes for the *PLAT1*, *PLAT2* and *PLAT3* genes were generated by PCR from cDNA with the primers, PLAT1-F probe and PLAT1-R probe, PLAT2-F probe and PLAT2-R probe, and PLAT3-F probe and PLAT3-R probe (Table S1 in File S1), respectively. The RT-PCR analysis was performed essentially as described before [37]. At least 10 seedlings per genotype were grown for 14 d on control plates before transfer to the respective stress and control medium. The optimal cycle number was determined for each primer pair (Table S2 in File S1). Expression values were corrected for the *ACTIN* and *UBIQUITIN* signal intensities and expressed as relative values compared to Col-0. For expression analysis, 3 independent experiments were performed.

### ABA determination

The extraction and analysis of ABA was carried out as described previously [39].

### PLAT1-YFP localisation

For transient *PLAT1-YFP* expression a single colony of *A. tumefaciens* LBA4404 containing either *PLAT1:PLAT1-YFP* or the *ER-rk CD3-959* ER-marker [40] construct was inoculated into 5 ml induction medium with antibiotics and grown overnight at 28°C. The bacteria were collected by centrifugation and resuspended in 10 mM MES and 10 mM MgCl_2_ containing 200 µM acetosyringone to an OD_600_ of 1.0. Aliquots (1 ml) of *A. tumefaciens* cells containing *PLAT1:PLAT1-YFP* and ER-marker construct were mixed together, and then a syringe was used to infiltrate the mixture into the lower surface of *N. benthamiana* leaves. YFP and mCherry fluorescence was visualized 48 h post infiltration, using Olympus confocal laser scanning microscope (model FV1000, Tokyo, Japan). For stable *PLAT-YFP* expression following ABA and salt treatment, the *PLAT1:PLAT1-YFP* line *YFP13-1* was employed, 10 plants per treatment.

### Transactivation assay

Suspensions of *A. tumefaciens* carrying the respective *35S:ABF_1–4_* overexpression constructs and the *PLAT1:PLAT1-GUS* rescue/reporter construct were mixed in a ratio of 1∶1. The resulting mixed suspensions were used to infiltrate leaves of 6-w-old greenhouse grown *N. benthamiana* in soil. As control, leaves were infiltrated with *A. tumefaciens* carrying the *PLAT1:PLAT1-GUS* rescue construct only, or 10 mM MgCl_2_. The GUS fluorometric assays were carried out as described previously [41]. Samples were isolated from the infiltrated regions 2 d after infiltration for 5 independent plants and ground in 500 µl of extraction buffer containing 50 mM sodium phosphate, 10 mM EDTA, 10 mM β-mercaptoethanol and 0.1% N-lauroylsarcosine (pH = 7.4). After centrifugation at 4°C for 10 min at 13,000 rpm, the supernatant was used for the determination of GUS enzyme activity. 50 µl of supernatants was transferred into one slot of a black 96-well plate and 50 µl of a 2 mM MUG (methylumbellifery-β-D-glucuronide, Sigma) solution was added. For each sample 3 technical replicates were measured. The samples were incubated at 37°C for 30, 60 and 90 min, before the reaction was stopped with 1 M sodium carbonate. A standard curve was prepared with MU (4-methylumbelliferone, Sigma) in a concentration range from 0 to 16 µM. Excitation was measured at 365 nm, emission at 455 nm. Total protein amount was determined by the Bradford assay. GUS enzyme activity was calculated in pmol Mu min^−1^ mg^−1^ protein.

### Expression and purification of ABFs

The ABFs were prepared employing a MBP-fusion purification procedure. Five ml of an overnight bacterial culture was incubated with 500 ml rich broth medium containing glucose and ampicillin. The cells were grown to 2×10^8^ cells ml^−1^ (A_600_ = 0.5). IPTG was added to a final concentration of 0.3 mM and a further incubation at 37°C for 2 h followed. The cells were harvested by centrifugation at 4,000 g for 20 min. The supernatant was discarded and the pellet re-suspended in 25 ml of column buffer (20 mM Tris-HCl, 200 mM NaCl, 1 mM EDTA, 1 mM DTT and 0.1 mM PMSF (pH = 7.4). The pellet was kept at −20°C overnight and thawed in cold water the next morning. The sample was placed in an ice-water bath and sonicated in short pulses of 15 s for at least 2 min. The suspension was centrifuged at 9,000 g for 30 min and the supernatant diluted 1/3 (v/v) with column buffer before purification. Amylase resin was poured in a 5 ml column and washed with 8 column volumes of column buffer. The diluted sample was loaded and slowly ran over the column. The column was then washed with 12 volumes of column buffer and the proteins subsequently eluted with column buffer containing 10 mM maltose. 10 to 15 fractions containing 2 ml each were collected. Proteins were checked via SDS-page.

### Electrophoretic mobility shift assay

For EMSA, the 200 bp *PLAT1* promoter, generated by PCR with the primers pPLAT1-F and pPLAT1-R (Table S1 in File S1) was used as positive probe. The mutated version lacking the 2 ABRE elements was generated in 2 steps by PCR using the primers WIP-F1 and WIP-R1, and WIP-F2 and WIP-R2. The 2 PCR fragments were joined to create the negative 200 bp probe WIP1. The 200 bp *PLAT1* promoter fragment was labelled with [γ-^32^P]ATP using T4 polynucleotide kinase (5′ end labelling). The reaction was incubated for 1 h at 37°C, purified and eluted with 10 mM Tris, pH = 8.0. The labelled probe was incubated 30 min at room temperature with 5 µg of the respective ABFx protein extracts alone, with 100 fold molar excess of “cold” specific competitor (200 bp *PLAT1* promoter), and with 100 fold molar excess of “cold” negative probe (WIP1), including poly-dIdC as nonspecific competitor. The EMSA samples were run on a 5% native poly-acrylamide gel (10×10 cm). Before loading the samples, the gel was pre-run 40 min at 80 V and 4°C and the samples were run at 120 V and 4°C. After electrophoresis, radioactivity was detected in the dried gel as described above. Functionality of the EMSA assay and ABFx preparation using the MBP fusion protein was proven using the ABF1 protein extract and the published [34] positive ABRE-F and ABRE-R, and negative mABRE-F and mABRE-R control primers (Table S1 in File S1).

### Statistical analysis

Standard deviations and average values were calculated in excel. Statistical significance for differences between treatments was analysed using the unpaired two sided Student's *t*-test in excel. ***, ** or * indicate statistical significance at p<0.001, p<0.01 or p<0.05, respectively.

### Accession numbers

The AGI locus identifiers for the *Arabidopsis* PLAT-plant-stress family members are: PLAT1, AT4G39730; PLAT2, AT2G22170 and PLAT3, AT5G65158.

## Results

### *PLAT1* expression is induced by abiotic stress conditions

**Figure 1:**
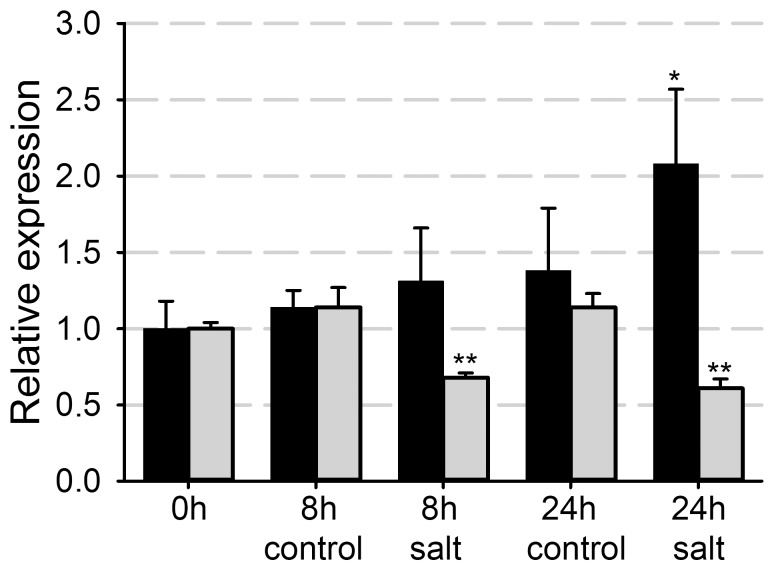
*PLAT1* expression is induced by salt stress conditions. Relative *PLAT1* and *PLAT2* expression in 14-d-old Col-0 seedlings following transfer to salt stress medium compared to control conditions. *PLAT1* black bars, *PLAT2* grey bars. Values are means of 3 replicates ± standard deviation. n≥10 per replicate. ** or * indicate statistical significance calculated using the unpaired Student's *t*-test at p<0.01 or p<0.05, respectively.

The PLAT-plant-stress subgroup of PLAT domain proteins comprises three *Arabidopsis* family members. Phylogenetic analysis of these *Arabidopsis* PLAT-plant-stress proteins using the neighbour-joining method showed that PLAT3 falls outside the other members (Figure S1A). *In silico* expression analysis by the eFP Browser [42] showed *PLAT1* and *PLAT2* expression during many developmental stages, which is affected under different stress conditions, whereas *PLAT3* is not represented on the Affymetrix ATH1 arrays. To corroborate these data, we investigated expression of the *PLAT* family members by northern blot and RT-PCR analysis. This showed that *PLAT1* was indeed expressed throughout development (Figure S1B). In contrast, *PLAT2* expression was only detected in young seedlings (Figure S1E), whereas *PLAT3* expression could not be detected in any of the analysed organs and developmental stages, neither by northern blot nor RT-PCR analysis at 35 cycles. *PLAT1* expression was induced both by salt, following one day of watering with 200 mM NaCl (Figure S1C), and cold treatment, following incubation of 3-w-old plants at 8°C for 2 d (Figure S1D). *PLAT1* expression was also induced following the transfer of young seedlings to medium with 200 mM NaCl (Figure 1). However, both the extent of *PLAT1* induction and the temporal dynamics were different from that following salt stress using older plants grown in soil (Figure S1C), probably because *PLAT1* expression is highest in young seedlings (Figure S1B). *PLAT2* expression was repressed following the transfer of young seedlings to medium with 200 mM NaCl (Figure 1). These results confirmed the *in silico* expression data and suggested that among the *Arabidopsis* PLAT family members mainly PLAT1 is involved in abiotic stress responses.

### PLAT1 promotes tolerance towards abiotic stress conditions

To analyse PLAT function in stress responses and plant development, we obtained T-DNA insertion mutants for *PLAT1* and *PLAT2.* Two independent homozygous *Arabidopsis* loss-of-function mutants were obtained for *PLAT1* (*plat1-1* and *plat1-2*) and one promoter insertion mutant was obtained for *PLAT2* (*plat2*) from the SALK and SAIL mutant collections, respectively [43], [44]. Since both *plat1* mutants exhibited similar phenotypic defects, only the characterisation of the *plat1-1* mutant is described in detail. The *plat1-1* and *plat2* mutants exhibited no obvious growth defects under control conditions and most likely represent null alleles because *PLAT1* and *PLAT2* expression was not detected in the respective insertion mutants (Figure S1E).

**Figure 2:**
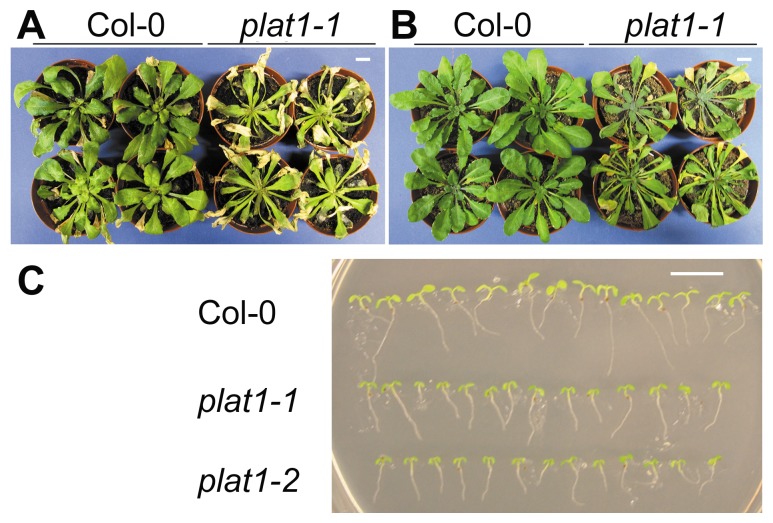
PLAT1 loss-of-function reduces abiotic stress tolerance. (**A**) Salt stress tolerance in wild-type (Col-0) and *plat1-1* seedlings irrigated with 200 mM NaCl for 14 d. n≥10 (**B**) Drought stress tolerance in wild-type and *plat1-1* seedlings, following 14 d without watering. n≥10 (**C**) Cold stress tolerance in 7-d-old wild-type, *plat1-1* and *plat1-2* seedlings following 14 d of incubation at 8°C. n = 14. Scale bar = 1 cm.

**Figure 3:**
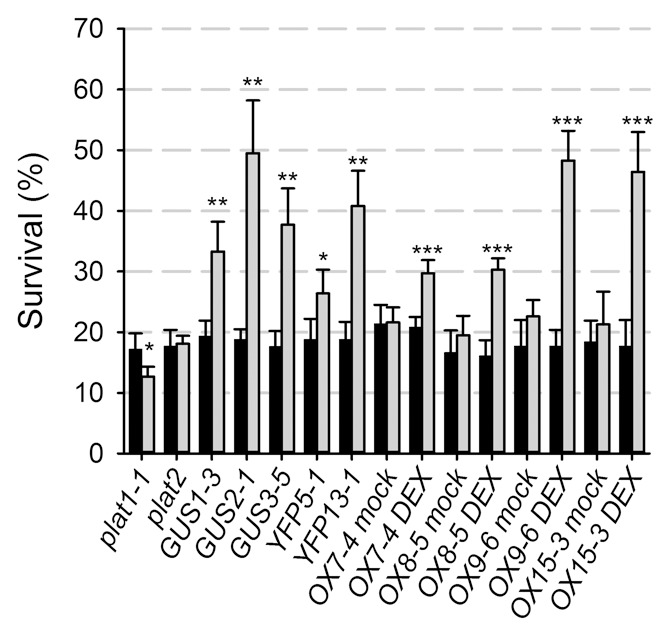
PLAT1 promotes tolerance towards salt stress conditions. Survival, expressed as the percentage of the seedlings transferred at the 6-d-old stage that developed (pale) green leaves during 4 d of salt stress conditions for the different mutant and transgenic lines (grey bars, n≥26 per replicate) compared to Col-0 control plants (black bars, n≥12 per replicate) that were grown on the same plates. Values are means of 3 replicates ± standard deviation. ***, ** or * indicate statistical significance calculated using the unpaired Student's *t*-test at p<0.001, p<0.01 or p<0.05, respectively.

The *plat1-1* mutant was more sensitive to salt and drought stress, as well as cold stress conditions (Figure 2), evident by a reduction in root length upon growth at 8°C from 9.41±1.25 mm for Col-0 to 7.02±1.27 mm or 6.20±0.94 mm for *plat1-1* and *plat1-2*, respectively (p<0.001, n = 14). In contrast, the *plat2* mutant did not show obvious changes in salt stress tolerance (Figure 3). Together with the differential effect of salt stress on expression of the *PLAT* family members (Figure 1), these data support that only PLAT1 is involved in abiotic stress tolerance.

To analyse whether PLAT1 also plays a role in biotic stress responses, we studied *PLAT1* expression following inoculation of plants with the hemibiotrophic pathogen *P. syringae* pv. *tomato* DC3000 or DC3000 *avrRpm1* (RPM1, Figure S2A), and the necrotrophic fungal pathogen *S. sclerotiorum* (Figure S2B). This showed that *PLAT1* expression was not specifically affected by these pathogens since increased expression was also observed for the respective control treatments (10 mM MgCl_2_ and PDA medium). Next, disease symptom development following inoculation with *P. syringae* pv. *tomato* DC3000 was investigated. Disease symptom development was not obviously affected in *plat1-1* (Figure S2C), which is in agreement with the fact that biotic stress conditions did not significantly affect *PLAT1* expression.

**Figure 4:**
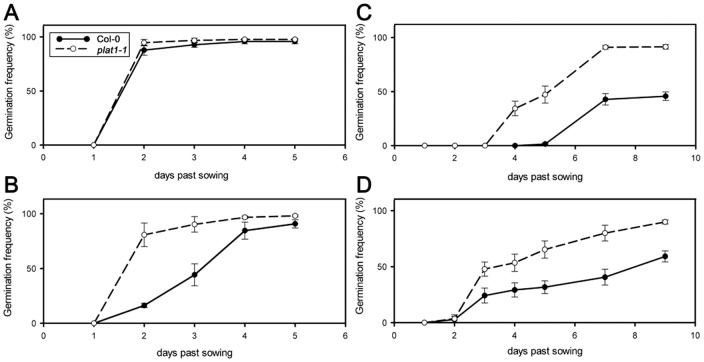
PLAT1 loss-of-function reduces ABA sensitivity during seed germination. (**A**) Seed germination of *plat1-1* and wild-type (Col-0). (**B**) Seed germination of *plat1-1* and wild-type on 300 mM mannitol (osmotic stress). (**C**) Seed germination of *plat1-1* and wild-type on 200 mM NaCl (salt stress). (**D**) Seed germination of *plat1-1* and wild-type on 1.5 µM ABA. Values are means of 3 replicates ± standard deviation. n≥100 per replicate.

Because ABA is strongly involved in abiotic stress responses, we analysed whether PLAT1 function is correlated with ABA signalling. One characteristic effect of ABA is the inhibition of seed germination [45]. Further, seed germination frequency is reduced by salt and osmotic stress conditions, resulting from increased ABA signalling. Under control conditions the germination frequency of the *plat1-1* seeds was similar to that of wild-type (Figure 4A). However, under osmotic stress using 300 mM mannitol (Figure 4B) and salt stress conditions employing 200 mM NaCl (Figure 4C), the *plat1-1* seeds exhibited a higher germination frequency compared to wild-type, suggesting that ABA signalling is reduced in *plat1-1*. Indeed, *plat1-1* seed germination is less severely reduced by ABA (1.5 µM) compared to wild-type (Figure 4D).

Since a change in seed dormancy could have influenced the seed germination assays and thus interpretation for ABA sensitivity, we analysed germination for the different PLAT lines on control medium without prior stratification. This showed that seed dormancy was only affected by PLAT2 loss-of-function (Figure S3). Further, ABA levels were not significantly affected in *plat1-1* plants compared to wild-type control (Table S3 in File S1). Together, these results indicate that PLAT1 loss-of-function reduced ABA sensitivity, which could have caused increased sensitivity towards abiotic stress conditions.

We generated genomic *PLAT1* rescue constructs, where the PLAT1 protein was fused to either the YFP or GUS reporter proteins. Following genetic complementation of the *plat1-1* mutant, seed responses towards ABA could be completely restored back to that of wild-type employing the genomic rescue/reporter constructs: 5 out of 6 lines for *PLAT1:PLAT1-GUS* (Figure S4A) and 15 out of 20 lines *PLAT1:PLAT1-YFP* (Figure S4B). This confirmed that the defects in abiotic stress tolerance were indeed caused by the T-DNA insertion in the *plat1-1* mutant and that the PLAT1 fusion proteins are functional in these rescued lines. Transgenic expression levels can be influenced to a large extend by plant sequences flanking the respective T-DNA integration sites, causing variation between individual transgenic lines harbouring the same T-DNA construct. In addition to transformed *plat1-1* lines with restored (wild-type) ABA sensitivity, also transgenics were obtained exhibiting increased ABA sensitivity and thus tissue specific *PLAT1* overexpression phenotype (Figure S4). To study whether *PLAT1* overexpression would confer an opposite phenotype compared to the *plat1* mutant, we selected these lines with tissue specific *PLAT1* overexpression for further analysis and also generated ectopic *PLAT1* overexpression lines. Because we could not obtain transgenics harbouring constitutive *PLAT1* overexpression, we generated lines with inducible *PLAT1* overexpression (*35S>>PLAT1*) employing the dexamethasone inducible *OP-LhGR* two component system [46]. From 17 transgenic *PLAT1* OX lines, the majority exhibited delayed germination on 1.5 µM ABA (Figure S4C).

To compare tissue specific with ectopic overexpression, which could cause considerable differences in overexpression phenotypes [47], we selected the *GUS1-3, GUS2-1, GUS3-5, YFP5-1* and *YFP13-1* genomic rescue lines with increased ABA sensitivity in the seed germination assays for further analysis. In these selected tissue specific (GUS and YFP) *PLAT1* overexpression lines, *PLAT1* transcript levels were increased to a similar extend as the ectopic (OX) overexpression lines, compared to wild-type control (Table S4 in File S1). The different types of *PLAT1* overexpression lines promoted salt stress tolerance after transfer to medium containing both 200 mM NaCl and 5 µM dexamethasone (DEX) (Figure 3). Thus, *PLAT1* overexpression resulted in higher *PLAT1* expression levels and reciprocal phenotypic effects compared to the *plat1-1* mutant, confirming that PLAT1 functions in promoting tolerance towards abiotic stress conditions.

### PLAT1 promotes growth

**Figure 5:**
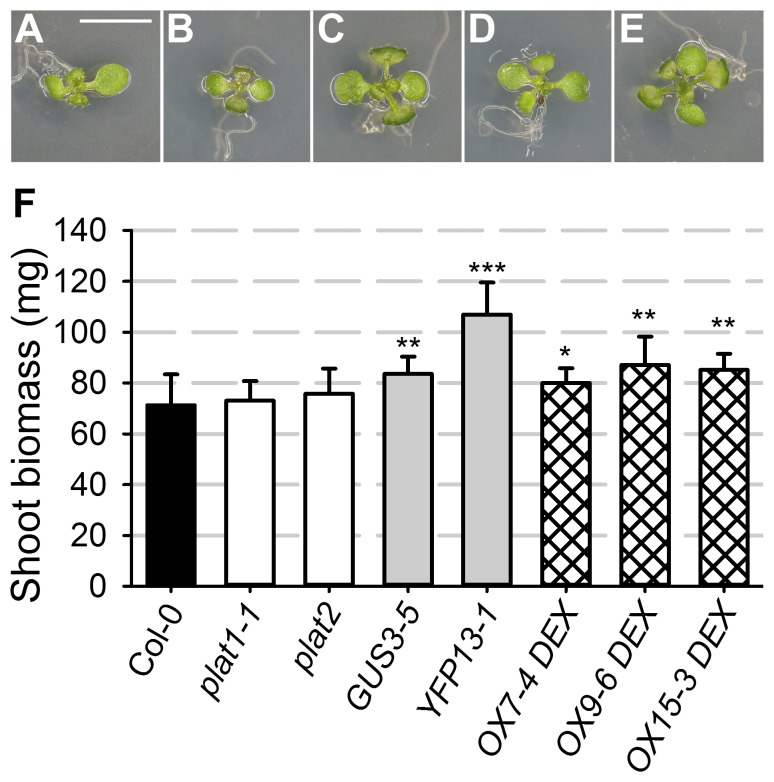
PLAT1 promotes plant growth. Phenotypes of plants from control medium including 5 µM DEX from the salt stress experiment shown in Figure 3. (**A**) Wild-type. (**B**) *plat1-1*. (**C**-**E**) Three independent *PLAT1* ectopic overexpression lines (OX). Scale bar = 1 cm. (**F**) Shoot biomass production (weight per 5 shoots) for the different PLAT1 lines. Values are means of 8 replicates ± standard deviation. ***, ** or * indicate statistical significance calculated using the unpaired Student's *t*-test at p<0.001, p<0.01 or p<0.05, respectively.

The DEX induced *PLAT1* OX lines from the salt stress experiment shown in Figure 3, appeared to show faster development on control medium including DEX (Figure 5A-E). Therefore, shoot and root growth was analysed in the *plat1-1* mutant and *PLAT1* overexpression lines. This showed that the different *PLAT1* overexpression lines produced more shoot biomass compared to wild-type control (Figure 5F), while the length of the root apical meristem and total root length were not affected (Table S5 in File S1). Interestingly, the number of emerged lateral roots was increased, but only for the ectopic *PLAT1* (OX) overexpression lines (Table S5 in File S1). This effect on lateral root formation was the only difference observed between the tissue specific and ectopic overexpression lines, and could reflect the difference between increased expression at its natural expression site (GUS and YFP) and broader expression in new (ectopic) cell types (OX). In contrast, PLAT1 loss-of-function did not affect either shoot or root growth, except for a reduced number of emerged lateral roots (Figure 5F, Table S5 in File S1).

### *PLAT1* is expressed in structures related to the regulation of water household

**Figure 6:**
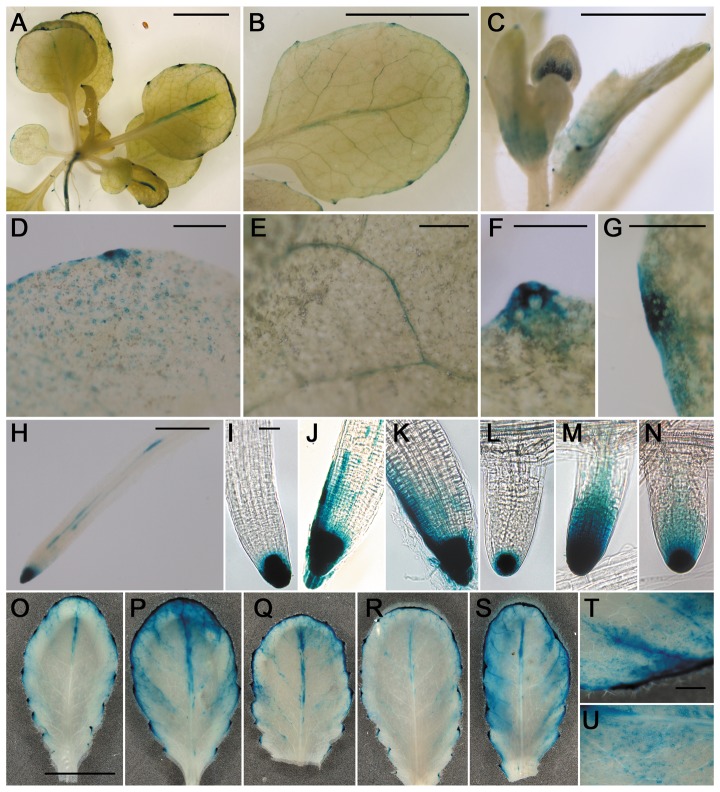
*PLAT1* is expressed in the vasculature, hydathodes and stomata of aerial organs. *PLAT1* expression is reflected by GUS activity in *plat1-1* mutant plants rescued by the *PLAT1:PLAT1-GUS* rescue/reporter construct (line *GUS3-5*). The *PLAT1* promoter confers expression in the leaf vasculature (**A**, **B**, **E**), hydathodes (**B**, **F**, **G**), floral organs (**C**), stomata (**D**) and the primary root tip and root pericycle cells (**H**). *PLAT1* expression in primary root tips, 24 h following transfer to control (**I**), 200 mM NaCl (**J**) or 1.5 µM ABA plates (**K**). *PLAT1* expression in lateral root tips, 24 h following transfer to control (**L**), 200 mM NaCl (**M**) or 1.5 µM ABA plates (**N**). *PLAT1* expression in fully expanded rosette leaf from 4-w-old seedling, control (**O**), following 24 h (**P**), or 48 h (**Q**) of treatment with 1.5 µM ABA and following 24 h (**R**), or 48 h (**S**) watering with 200 mM NaCl. Detail of rosette leaf showing expansion of expression domain in leaf mesophyll following 24 h ABA treatment (**T**), or 48 h watering with NaCl (**U**). Scale bar = 1 cm (A-C) and (O-S), 1 mm (D-H), (T), (U), 0.1 mm (I-N). n≥10.

The *PLAT1:PLAT1-GUS* rescue/reporter construct conferred GUS activity (4 out of 6 lines analysed) in vascular tissue, leaf edges, hydathodes and stomata, which are structures related to the regulation of water household. The expression in stomata corresponds to the identification of PLAT1 in guard cells [48]. GUS activity could also be observed in floral organs, root tips, pericycle cells and lateral root primordia (Figure 6A-6H). The basal *PLAT1* expression pattern in the shoot correlated with the abiotic stress tolerance, while that of the root correlated with the changes in lateral root number for the *plat1-1* mutant and *PLAT1* overexpression lines. Both treatment with salt and ABA resulted in an expanded *PLAT1* expression domain in root tips (Figure 6I-6N) and leaves (Figure 6O-6U), evident by the GUS activity in leaf mesophyll cells (Figure 6T and 6U), as well as in root pericycle cells (Figure S5).

**Figure 7:**
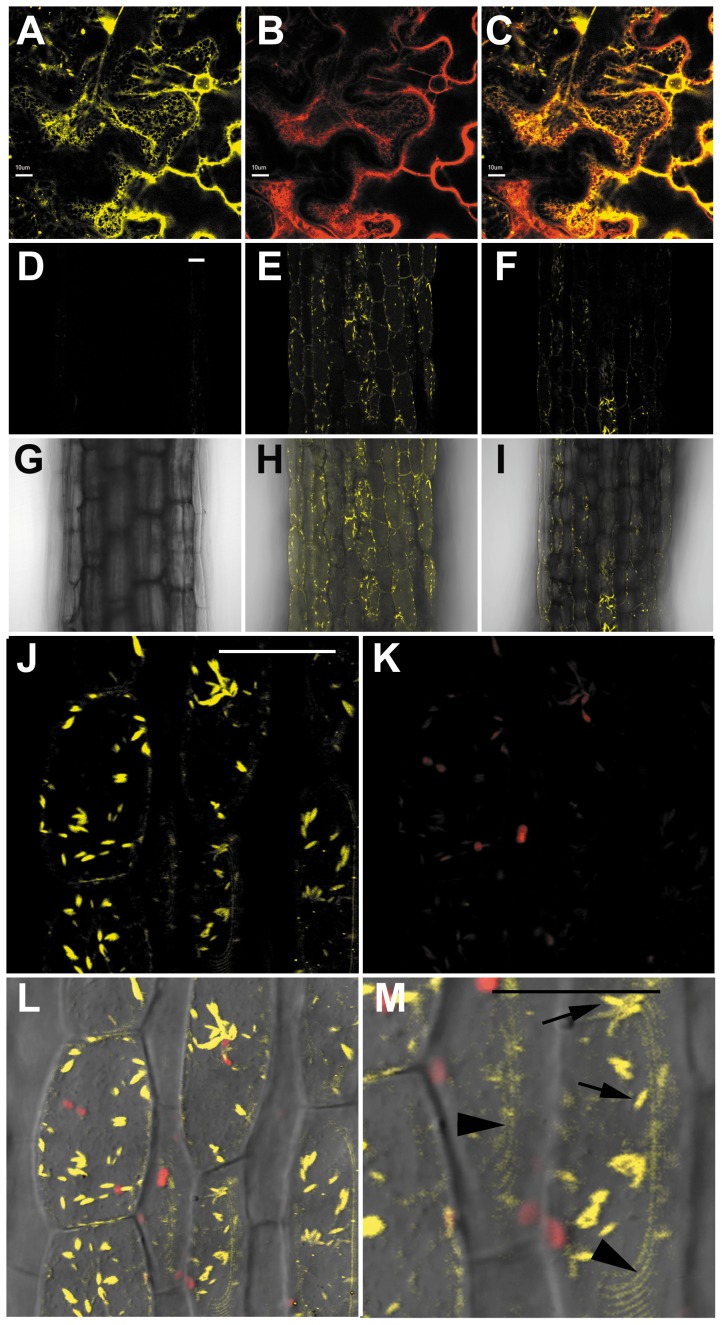
PLAT1 is localised to the ER in *Arabidopsis* and *Nicotiana benthamiana*. Transient transformation of the *PLAT1:PLAT1-YFP* construct to *N. benthamiana*. YFP channel showing *PLAT1-YFP* expression (**A**), RFP channel showing *ER-rk CD3-959* mCherry marker expression (**B**), co-localisation of PLAT1-YFP with the ER-rk CD3-959 marker (C). *PLAT1* expression in stable *plat1-1* transgenics (line *YFP13-1*) rescued by the *PLAT1:PLAT1-YFP* rescue/reporter construct (D-I) 48 h following transfer of 3-d-old seedlings to control, NaCl or ABA plates, with YFP channel (D-F) and merged YFP and bright field channels (G-I). *PLAT1* expression following transfer to control medium (**D**, **G**), following transfer to 200 mM NaCl (**E**, **H**), and expression following transfer to 1.5 µM ABA (**F**, **I**). Hypocotyl section from line *YFP13-1* with YFP channel showing *PLAT1:PLAT1-YFP* reporter activity (**J**), red autofluorescence of chloroplasts (**K**), merged image of bright field, YFP and red autofluorescence (**L**). (**M**) Detail of (L), showing PLAT1 localisation to putative ER bodies (arrows) and the ER (arrow heads). Scale bar = 10 µm (A-C), 0.1 mm (D-L), 0.05 mm (M). n≥10.

To understand protein function, knowledge on its sub-cellular localisation is essential, because this influences access to and availability of interaction partners [49]. According to the Aramemnon membrane protein database [50], PLAT1 was predicted to contain a signal peptide involved in the secretory pathway and one transmembrane spanning domain. To analyse the sub-cellular localisation we transiently transformed the *PLAT1:PLAT1-YFP* construct together with different organelle markers [40] to *N. benthamiana*, which showed that the PLAT1-YFP fusion protein co-localised with the mCherry ER-marker ER-rk CD3-959 (Figure 7A-7C). The analysis of YFP reporter activity in the stable rescued *plat1-1* transformants harbouring the *PLAT1:PLAT1-YFP* construct reflected the expression pattern evident from the GUS rescue reporter lines, confirmed *PLAT1* induction by salt and ABA (Figure 7D-7I), and PLAT1 localisation to the ER (Figure 7J-7M). Further, PLAT1 is localised to rod shaped ER structures that resemble ER bodies (Figure 7M).

### The *PLAT1* promoter is a direct target of the ABF transcription factors

**Figure 8:**
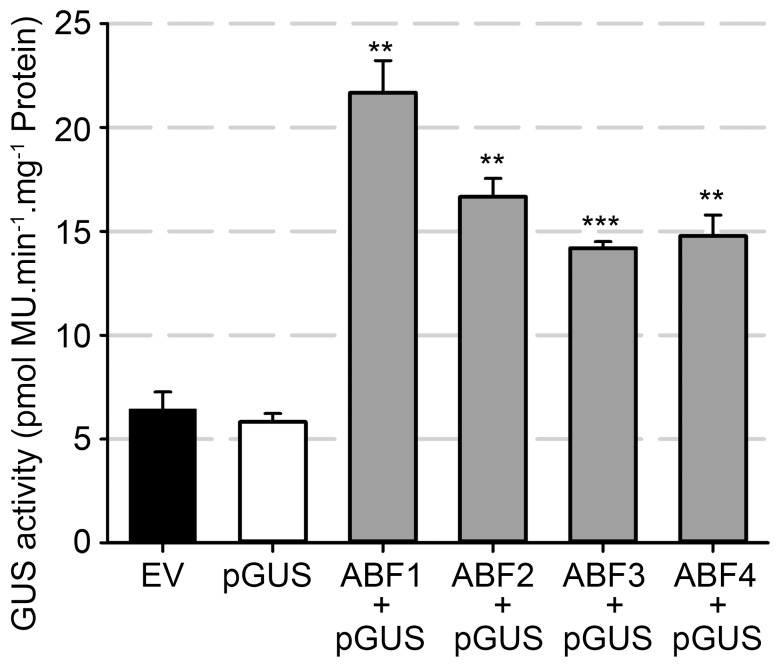
The *PLAT1* promoter is activated by the ABF transcription factors. GUS activity in 8-w-old *N. benthamiana* leaves infiltrated with *A. tumefaciens* harbouring the *PLAT1:PLAT1-GUS* reporter (pGUS) or an empty vector (EV) as negative control, compared to the simultaneous infiltration of the *35S:ABF_1–4_* and *PLAT1:PLAT1-GUS* constructs. Values are means of 3 replicates ± standard deviation. *** or ** indicate statistical significance calculated using the unpaired Student's *t*-test at p<0.001 or p<0.01, respectively.

Sequence analysis of the *PLAT1* promoter identified 2 G-boxes/ABRE elements (CACGTG motif) located at positions −165 to −156 and −134 to −126 relative to the transcription start site. ABRE elements are direct binding sites of the ABF/AREB (bZIP) transcription factors, which are positive mediators of the ABA signalling pathway [34], [35]. We employed a transactivation assay to investigate whether the *PLAT1* promoter is indeed regulated by these ABF transcription factors. Leaves from wild-type tobacco *N. benthamiana* plants were simultaneously infiltrated with *A. tumefaciens* harbouring either one of the *35S:ABF_1–4_* overexpression constructs (ABF_1–4_) and the *PLAT1:PLAT1-GUS* genomic rescue/reporter construct (pGUS). Each of the double infiltrations resulted in significantly higher GUS activity levels, compared to infiltration with the *PLAT1:PLAT1-GUS* reporter construct only (Figure 8). This indicated that the ABF transcription factors activate the *PLAT1* promoter.

Electrophoretic mobility shift assays (EMSA) confirmed these transactivation results and showed that the ABF1, ABF3 and ABF4 transcription factors directly bind to the *PLAT1* 200 bp promoter fragment (−200 to +1), which contains the 2 ABRE elements. This ABF binding was specifically competed with a 100-fold molar excess of unlabelled 200 bp *PLAT1* promoter fragment (PLAT1; Figure S6), but not with the mutated 200 bp promoter fragment WIP1, where point mutations were introduced in the two ABRE elements (WIP1; Figure S6). Thus, *PLAT1* expression is regulated by the ABA signalling pathway as direct target of the ABF transcription factors.

### PLAT1 promotes tolerance towards ER stress elicited by tunicamycin

**Figure 9:**
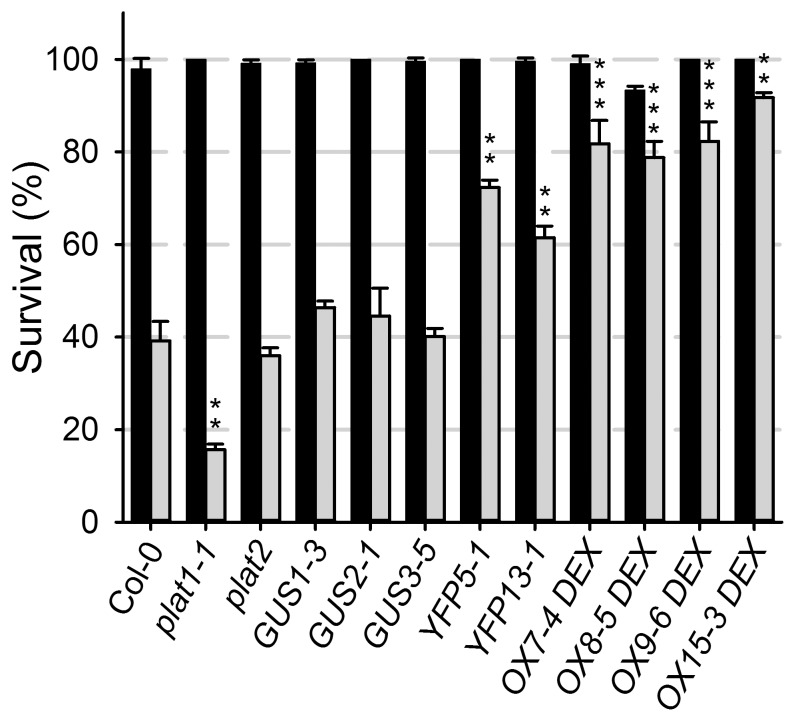
PLAT1 promotes tolerance towards tunicamycin elicited ER stress. Survival is expressed as the percentage of the plated seeds that developed (pale) green seedlings. Control conditions black bars, ER stress (0.05 µg l^−1^ TM) grey bars. Values are means of 3 replicates ± standard deviation. n≥70 per replicate. *** or ** indicate statistical significance calculated using the unpaired Student's *t*-test at p<0.001 or p<0.01, respectively.

The PLAT1 subcellular localisation in the ER (Figure 7) suggested that PLAT1 function is related to ER stress. Under stress conditions, misfolded proteins accumulate in the ER that eventually cause ER stress [51], resulting in reduced growth and induction of the unfolded protein response (UPR) to compensate for this increased accumulation of misfolded proteins. TM is used as ER stress agent and interferes with N-linked glycosylation of secreted glycoproteins, which prevents protein folding in the ER [51]. The tolerance of the different *PLAT1* overexpression lines towards TM was improved compared to Col-0 wild-type (Figure 9). In contrast, the *plat1-1* mutant was more sensitive, while the response towards TM was not affected in the *plat2* mutant (Figure 9), which supports a function for PLAT1 in ER stress responses and/or UPR. The basal expression levels for the ER stress markers *BIP1,2*, *CNX1*, *CRT1* and *PDIL* [52] was higher for the different *PLAT1* overexpression lines (Figure S7), which suggests that the capacity of these lines to deal with unprocessed and/or misfolded proteins in the ER is increased, which could contribute to an increased abiotic stress tolerance.

## Discussion

The complete *Arabidopsis* genome sequence is available, nevertheless only for a relatively low percentage of *Arabidopsis* proteins experimental evidence concerning their function is available. While among the PLAT-plant-stress family only *Ca*TIN1 function has been studied by heterologous expression in tobacco [4], for other family members only limited *in silico* expression data is available. This showed that these members were induced by different abiotic and biotic stimuli, suggesting that PLAT-plant-stress proteins in general could promote tolerance towards stress responses and thus can be of great importance for developing stress tolerant crops. Nevertheless, little information is available on the function of these proteins. We assigned a function to the previously uncharacterised *Arabidopsis* PLAT domain proteins, which are members of this PLAT-plant-stress subgroup. The combination of genetic and physiological approaches supported functional diversification within this *Arabidopsis* PLAT protein family. PLAT1 critically functions as positive regulator of abiotic stress tolerance and also confers increased plant growth. Because *PLAT1* is a direct activated target of the ABF transcription factors and PLAT1 levels affect ABA sensitivity based on seed germination assays, *PLAT1* represents a novel downstream target of the ABA signalling pathway. PLAT2 appears to function specifically in seed dormancy, while *PLAT3* might represent a non-expressed pseudogene.

Analysis of the *plat1-1* loss-of-function mutant, *PLAT1* overexpression lines and genetic complementation of the *plat1-1* mutant, showed that PLAT1 critically functions as positive regulator of abiotic stress tolerance. Abiotic stress treatments induced *PLAT1* expression, PLAT1 loss-of-function resulted in reduced abiotic stress tolerance, whereas *PLAT1* overexpression conferred an inverse response, evident from increased cold, drought and salt stress tolerance. No change in pathogen susceptibility was detected for the *plat1-1* loss-of-function mutant, which correlates with the fact that pathogen infection did not significantly affect *PLAT1* expression. It is however possible that an altered pathogen resistance in *plat1-1* could have been too weak to be detected in the employed pathosystem. Otherwise, functional redundancy among the PLAT family members (partially) could have compensated for PLAT1 loss-of-function in biotic stress tolerance.

High ABA levels confer resistance towards abiotic stress conditions like drought and salinity, which is linked to its role in regulating stomatal aperture, while ABA shows antagonistic interaction with pathogen defence signalling pathways [26], [28]–[31]. The ABF/ABRE transcription factors, which are positive mediators of the ABA signalling pathway [34], [35], were shown to be induced by cold (ABF1), salinity (ABF2 and ABF3) and drought stress (ABF4) [34], while *ABF2* or *ABF3* overexpression enhanced abiotic stress tolerance [53]. *PLAT1* overexpression increased and loss-of-function reduced sensitivity towards ABA during seed germination. Because ABA levels were not significantly affected in the different PLAT1 lines, *PLAT1* overexpression resembled the effect of increased ABA signalling, including the differential regulation of abiotic and biotic stress tolerance.

Although it is widely speculated that the effect of ABA signalling on stress tolerance in higher plants is regulated by the complex (antagonistic) interactions with other phytohormones, our understanding of the ABA signalling pathway leading to the adaptation of naturally occurring multi-stress responses remains unclear [54]. Genes harbouring two ABRE elements in their promoter were predicted to be direct (activated) target genes of the ABF/ABRE transcription factors [34] and the expression for a large number of such genes was affected by a triple ABF loss-of-function mutant [35]. However, only the *DREB2C* and *RD29B* genes were functionally shown to be direct ABF target genes [55], [56]. The *PLAT1* promoter harbours two ABRE elements, located at positions −165 to −156 and −134 to −126 relative to the transcription start site. In agreement with these findings, transactivation and EMSA experiments showed that the ABF transcription factors directly bind to and activate the *PLAT1* promoter. Analysis of *plat1-1* lines complemented with the *PLAT1:PLAT1-GUS* rescue/reporter construct showed that *PLAT1* is expressed in the leaf vasculature, hydathodes and stomata, which are structures linked to the regulation of water household, but also at specific regions in the root. Together with the induced *PLAT1* expression levels as well as expanded expression domain following ABA treatment and abiotic stress conditions, *PLAT1* expression correlates with PLAT1 function in abiotic stress tolerance and its regulation by ABA signalling. Together, these data showed that *PLAT1* represents a novel component of the ABA signalling as direct target of the ABF transcription factors, which might explain its function in stress tolerance.

ABA in general negatively affects plant growth, mainly through crosstalk with the brassinosteroid pathway [57], and promotes quiescence of stem cells resulting in reduced root growth and lateral root formation [58]–[60]. In addition to the effects on stress tolerance, which could be correlated with *PLAT1* being a downstream target of the ABA signalling pathway, PLAT1 promotes plant growth, evident by a faster development and consequently increased shoot biomass. Therefore, PLAT1 function exhibits both expected and unexpected ABA related responses. Heterologous *CaTIN1* overexpression in tobacco also resulted in increased abiotic stress tolerance and plant growth, but additionally conferred increased biotic resistance, probably through influencing the redox state. Further, *CaTIN1* expression was induced by ethylene treatment and infection by tobacco mosaic virus, but not following ABA treatment [4]. Thus, despite the fact that PLAT1 and *Ca*TIN1 are orthologs, partially convergent evolution occurred in the different plant species on protein function and transcriptional regulation.

Transient expression experiments and analysis of *plat1-1* lines complemented with the *PLAT1:PLAT1-YFP* rescue/reporter construct showed that PLAT1 is localised to the ER, but also in rod shaped structures resembling ER bodies. This is supported by the induction of these structures with PLAT1-YFP signals following ABA treatment or salt stress conditions. ER bodies are specific to *Brassicaceae* and induced following stress conditions and wounding [61]–[63], but no direct correlation between ER bodies and abiotic stress responses has been shown. PLAT1 promotes tolerance towards the ER stress elicitor TM and the basal expression levels of ER stress markers, representing chaperonins functioning in ER stress and/or UPR.

Together our results indicate that PLAT1 functions in abiotic stress tolerance, either directly through promoting abiotic stress responses, and/or indirectly through improving basal tolerance/fitness. A direct promotion of abiotic stress responses could result from promoting ABA signaling, which is related to its function as novel direct target gene of the ABA signaling pathway. An indirect effect could result from stimulating ER stress responses for a higher basal tolerance/fitness. ER stress responses were shown to be indispensable for abiotic stress responses [64], [65] and the ER appears to play a prominent role in ABA-mediated stress signalling since ABA release from the ER is important for plants coping with stress [66]. The PLAT1 protein essentially harbours one transmembrane spanning domain and one large PLAT domain that covers the rest of the protein sequence and which has been shown to function in protein interaction. Therefore, PLAT1 most likely does not possess enzymatic activity, but rather functions as ‘docking site’ for interacting proteins with enzymatic or signalling activity functioning in ABA regulated pathways, enabling PLAT1 to regulate their activity.

To obtain plants through biotechnology or breeding approaches with increased tolerance towards adverse conditions, but without yield penalties under optimal growth conditions, it is important to identify all genes involved in stress responses and understand their function. Therefore, the identification and assignment of a function to the previously uncharacterised PLAT-plant-stress family member PLAT1 contributes to this important goal. The improved plant growth associated with the increased tolerance towards cold, drought and salt stress mediated by *PLAT1* overexpression could be an important asset in crop improvement. To enable the application of PLAT1 or other members from the PLAT-plant-stress family in crop improvement, future studies will be needed to address the multifaceted role of these proteins in stress tolerance and plant development.

## Supporting Information

Figure S1 ***PLAT1*** **expression patterns under different conditions.** (**A**) Phylogenetic tree of the PLAT-plant-stress subgroup. Phylogenetic analysis was carried out using the neighbour-joining method with 100 bootstraps and displayed using TreeTop. *Glycine max* (Glyma), *Zea mays* (GRMZM), *Oryza sativa* (Os), *Populus trichocarpa* (POPTR), *Sorghum bicolor* (Sb). (**B**) *PLAT1* expression in different organs from 3-w-old, 6-w-old and 12-w-old wild-type (Col-0) plants: F, Flower; H, hypocotyl; L, leaf; R, root; S, inflorescence stem and W, whole plant. (**C**) *PLAT1* expression following salt treatment (right) compared to control watering (left). (**D**) *PLAT1* expression following cold treatment. (**E**) *PLAT1* and *PLAT2* expression by RT-PCR in the respective T-DNA insertion mutants *plat1-1* and *plat2*. Bottom panels, rRNA for loading control (A-D).
(TIF)

Figure S2 **PLAT1 loss-of-function does not affect biotic stress tolerance.** (**A**) *PLAT1* expression following leaf infiltration of 10^7^ cfu ml^−1^ of *P. syringae* pv. *tomato* DC3000 or DC3000 *avrRpm1* in 10 mM MgCl_2_, compared to control treatment (MgCl_2_). (**B**) *PLAT1* expression following infection with *S. sclerotiorum* compared to control treatment (PDA). (**C**) Leaves from wild-type (Col-0) plants (Top panel) and *plat1-1* plants (Bottom panel), 3 d after infection with 10^7^ cfu ml^−1^ *P. syringae* pv. *tomato* DC3000. Scale bar = 1 cm, n≥10.
(TIF)

Figure S3 **PLAT2 functions in seed dormancy.** Seed germination of *plat1-1*, *PLAT1:PLAT1-GUS* line *GUS3-5*, *35S>>PLAT1* line *OX9-6*, *plat2* and wild-type (Col-0) on control medium including 5 µM DEX without prior stratification. Values are means of 3 replicates ± standard deviation. n≥100 per replicate.
(TIF)

Figure S4 **Increased ABA sensitivity by tissue specific or ectopic** ***PLAT1*** **overexpression.** (**A**, **B**) Seed germination of *plat1-1*, wild-type (Col-0) and *plat1-1* lines complemented with the *PLAT1:PLAT1-GUS* rescue construct (GUS) (A), or *plat1-1* lines complemented with the *PLAT1:PLAT1-YFP* rescue construct (YFP) (B) on medium supplemented with 1.5 µM ABA. (**C**) Seed germination of wild-type and transgenic lines harbouring the *35S>>PLAT1* ectopic overexpression construct (OX) on medium supplemented with 1.5 µM ABA and 5 µM DEX. Values are means of 3 replicates ± standard deviation. n≥100 per replicate.
(TIF)

Figure S5 ***PLAT1*** **expression is induced in adult roots by ABA treatment and salt stress.** *PLAT1:PLAT1-GUS* seedlings were monitored for *PLAT1* expression 8 h (A-C) and 24 h (D-F) following transfer to control, NaCl or ABA plates. (**A**, **D**) Detail of 2-w-old adult root with *PLAT1* expression in emerging lateral root primordia following transfer to control medium. (**B**, **E**) Detail of adult root with expanded expression domain following transfer to 200 mM NaCl. (**C**, **F**) Detail of adult root with expanded expression domain following transfer to 1.5 µM ABA. Scale bar = 0.1 mm, n≥10.
(TIF)

Figure S6 **PLAT1 functions as direct ABF target in ABA signalling.** EMSA assay showing that the ABF transcription factors bind to the 200 bp *PLAT1* promoter region containing two ABRE elements PLAT1 (*). This binding was specifically competed with a 100 molar excess of unlabelled *PLAT1* promoter fragment (PLAT1), but not the negative probe lacking the two ABRE elements (WIP1). Arrowhead indicates shifted band. Bracket indicates free probe.
(TIF)

Figure S7 ***PLAT1*** **overexpression lines exhibit higher basal expression levels for ER stress markers.** Relative expression levels for ER stress markers in the different *PLAT1* overexpression lines compared to wild-type (Col-0) and the *plat1-1* mutant. (**A**) *BIP1,2* (*HSP70*), (**B**) *CNX1* (*CALNEXIN1*), (**C**) *CRT1* (*CALRETICULIN1*) and (**D**) *PDIL* (*PROTEIN DISULFIDE ISOMERASE*-like). Values are means of 3 replicates ± standard deviation. n≥10 per replicate. ***, ** or * indicate statistical significance calculated using the unpaired Student's *t*-test at p<0.001, p<0.01 or p<0.05, respectively.
(TIF)

File S1 **Combined file containing supporting tables. Table S1**: List of primers used for cloning, genotyping of T-DNA insertion mutants and EMSA controls. **Table S2**: List of primers used for RT-PCR. **Table S3**: ABA levels are not affected by PLAT1. **Table S4**: *PLAT1* transcript levels in the different overexpression lines. **Table S5**: PLAT1 promotes lateral root formation.
(DOC)
