# Supplementary figures and images for: The Arabidopsis PLAT Domain Protein1 Is Critically Involved in Abiotic Stress Tolerance
Source: PLoS One. 2014 Nov 14;9(11):e112946. doi: 10.1371/journal.pone.0112946 (PMC4232524; doi:10.1371/journal.pone.0112946)

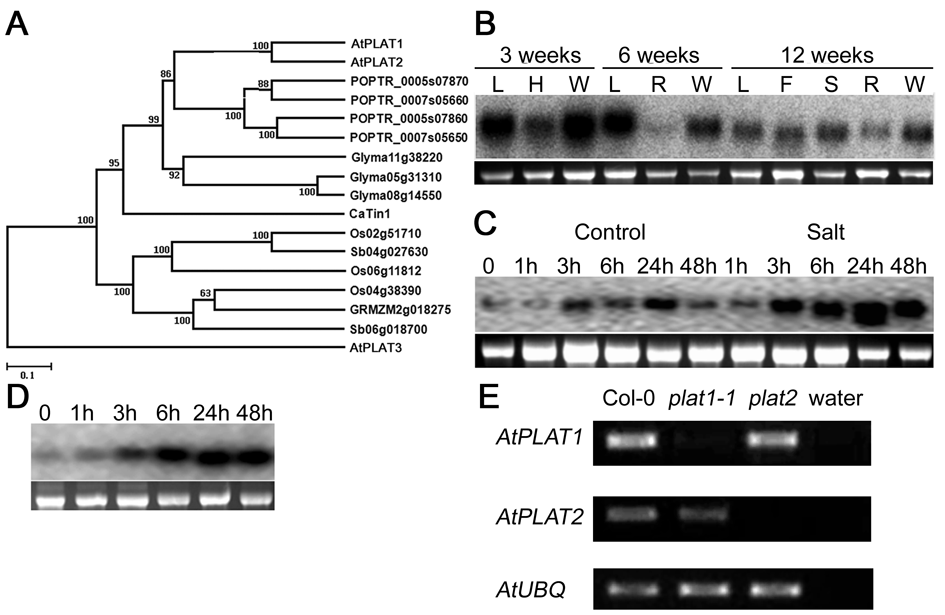

Supplement: Figure S1 — PLAT1 expression patterns under different conditions. (A) Phylogenetic tree of the PLAT-plant-stress subgroup. Phylogenetic analysis was carried out using the neighbour-joining method with 100 bootstraps and displayed using TreeTop. Glycine max (Glyma), Zea mays (GRMZM), Oryza sativa (Os), Populus trichocarpa (POPTR), Sorghum bicolor (Sb). (B) PLAT1 expression in different organs from 3-w-old, 6-w-old and 12-w-old wild-type (Col-0) plants: F, Flower; H, hypocotyl; L, leaf; R, root; S, inflorescence stem and W, whole plant. (C) PLAT1 expression following salt treatment (right) compared to control watering (left). (D) PLAT1 expression following cold treatment. (E) PLAT1 and PLAT2 expression by RT-PCR in the respective T-DNA insertion mutants plat1-1 and plat2. Bottom panels, rRNA for loading control (A-D). (TIF) [file pone.0112946.s001.tif]

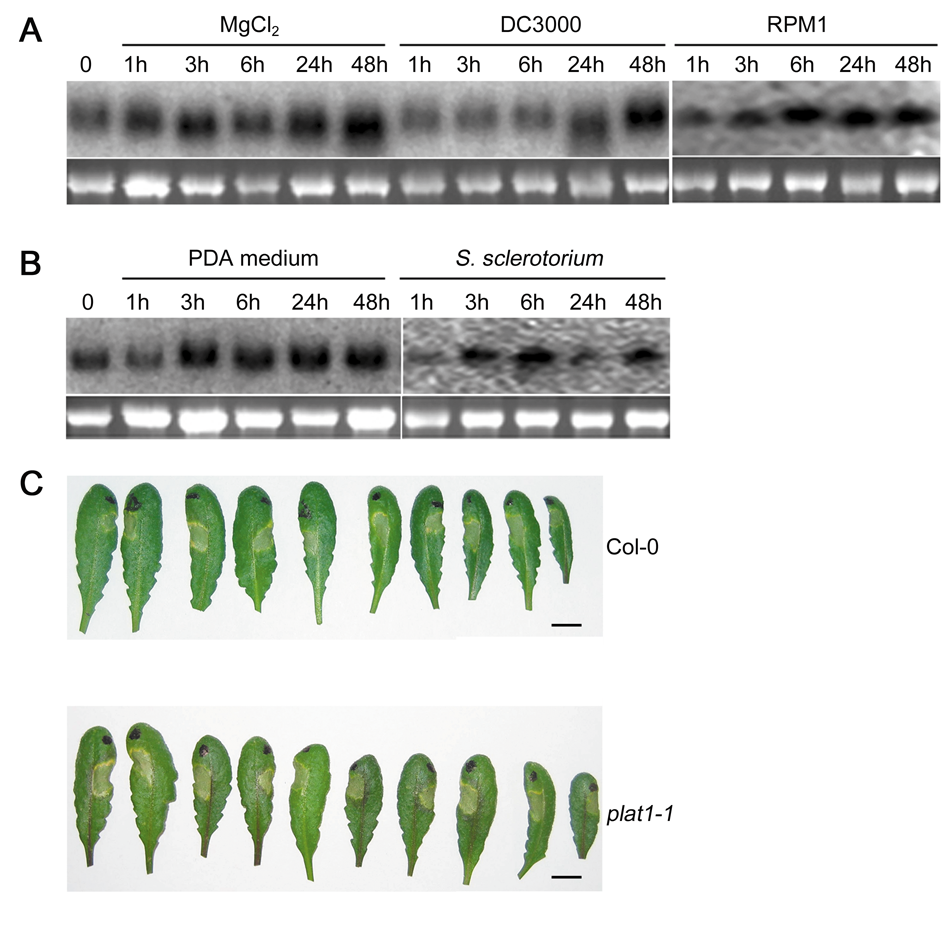

Supplement: Figure S2 — PLAT1 loss-of-function does not affect biotic stress tolerance. (A) PLAT1 expression following leaf infiltration of 107 cfu ml−1 of P. syringae pv. tomato DC3000 or DC3000 avrRpm1 in 10 mM MgCl2, compared to control treatment (MgCl2). (B) PLAT1 expression following infection with S. sclerotiorum compared to control treatment (PDA). (C) Leaves from wild-type (Col-0) plants (Top panel) and plat1-1 plants (Bottom panel), 3 d after infection with 107 cfu ml−1 P. syringae pv. tomato DC3000. Scale bar = 1 cm, n≥10. (TIF) [file pone.0112946.s002.tif]

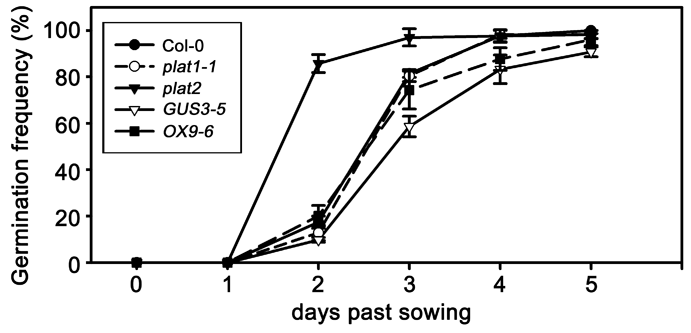

Supplement: Figure S3 — PLAT2 functions in seed dormancy. Seed germination of plat1-1, PLAT1:PLAT1-GUS line GUS3-5, 35S>>PLAT1 line OX9-6, plat2 and wild-type (Col-0) on control medium including 5 µM DEX without prior stratification. Values are means of 3 replicates ± standard deviation. n≥100 per replicate. (TIF) [file pone.0112946.s003.tif]

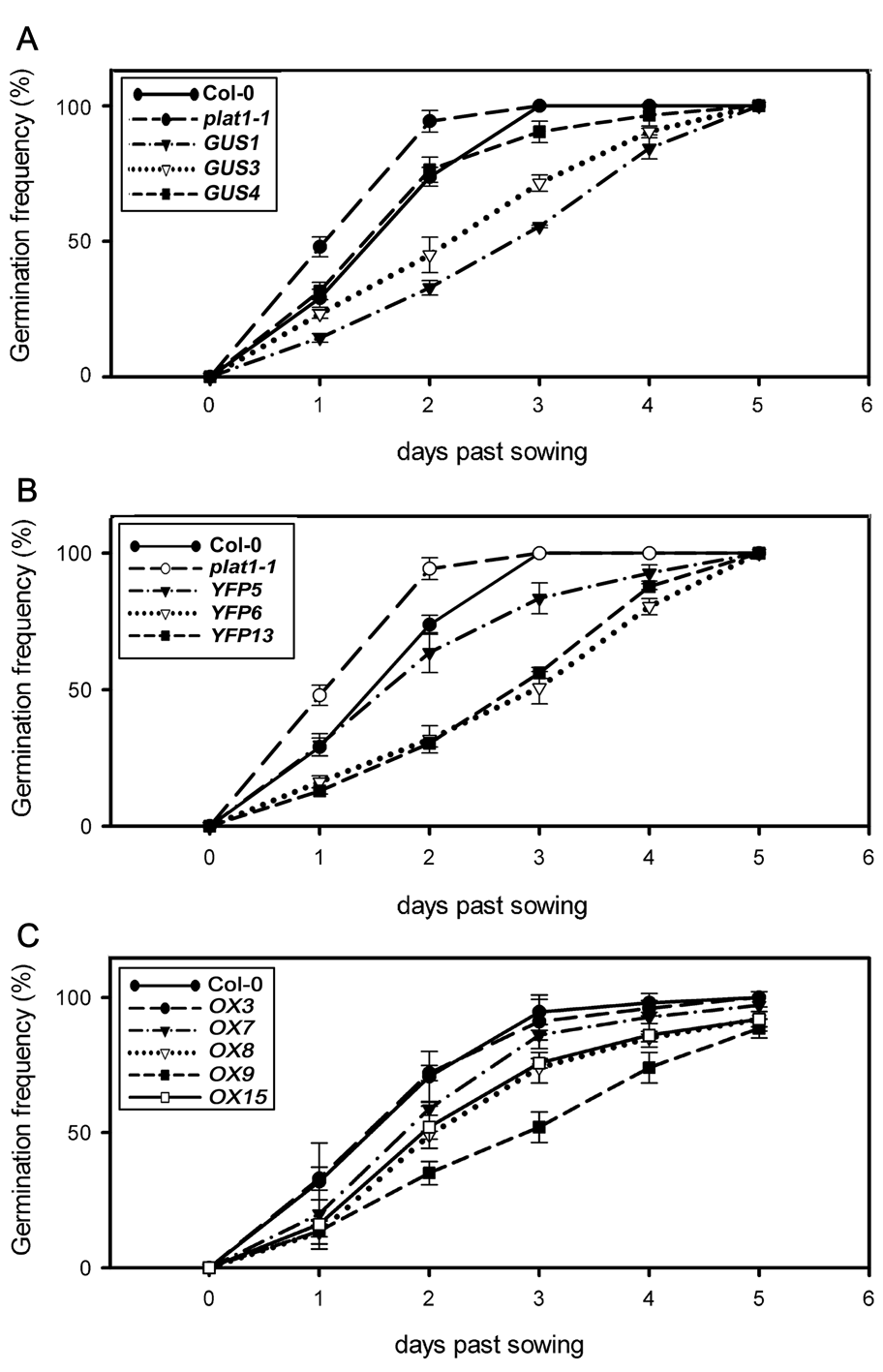

Supplement: Figure S4 — Increased ABA sensitivity by tissue specific or ectopic PLAT1 overexpression. (A, B) Seed germination of plat1-1, wild-type (Col-0) and plat1-1 lines complemented with the PLAT1:PLAT1-GUS rescue construct (GUS) (A), or plat1-1 lines complemented with the PLAT1:PLAT1-YFP rescue construct (YFP) (B) on medium supplemented with 1.5 µM ABA. (C) Seed germination of wild-type and transgenic lines harbouring the 35S>>PLAT1 ectopic overexpression construct (OX) on medium supplemented with 1.5 µM ABA and 5 µM DEX. Values are means of 3 replicates ± standard deviation. n≥100 per replicate. (TIF) [file pone.0112946.s004.tif]

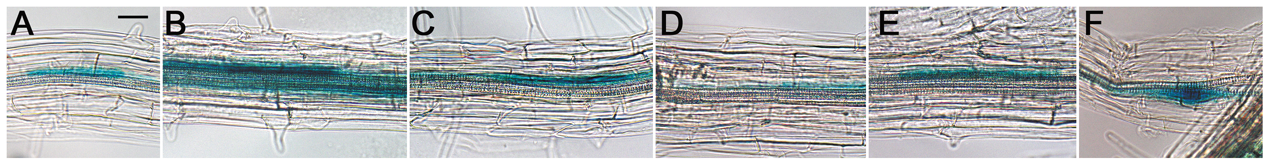

Supplement: Figure S5 — PLAT1 expression is induced in adult roots by ABA treatment and salt stress. PLAT1:PLAT1-GUS seedlings were monitored for PLAT1 expression 8 h (A-C) and 24 h (D-F) following transfer to control, NaCl or ABA plates. (A, D) Detail of 2-w-old adult root with PLAT1 expression in emerging lateral root primordia following transfer to control medium. (B, E) Detail of adult root with expanded expression domain following transfer to 200 mM NaCl. (C, F) Detail of adult root with expanded expression domain following transfer to 1.5 µM ABA. Scale bar = 0.1 mm, n≥10. (TIF) [file pone.0112946.s005.tif]

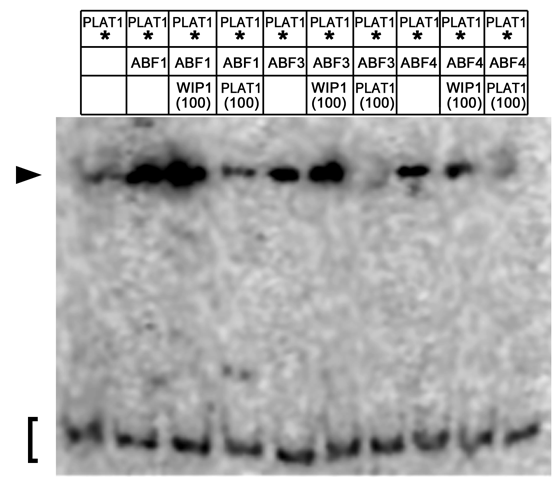

Supplement: Figure S6 — PLAT1 functions as direct ABF target in ABA signalling. EMSA assay showing that the ABF transcription factors bind to the 200 bp PLAT1 promoter region containing two ABRE elements PLAT1 (*). This binding was specifically competed with a 100 molar excess of unlabelled PLAT1 promoter fragment (PLAT1), but not the negative probe lacking the two ABRE elements (WIP1). Arrowhead indicates shifted band. Bracket indicates free probe. (TIF) [file pone.0112946.s006.tif]

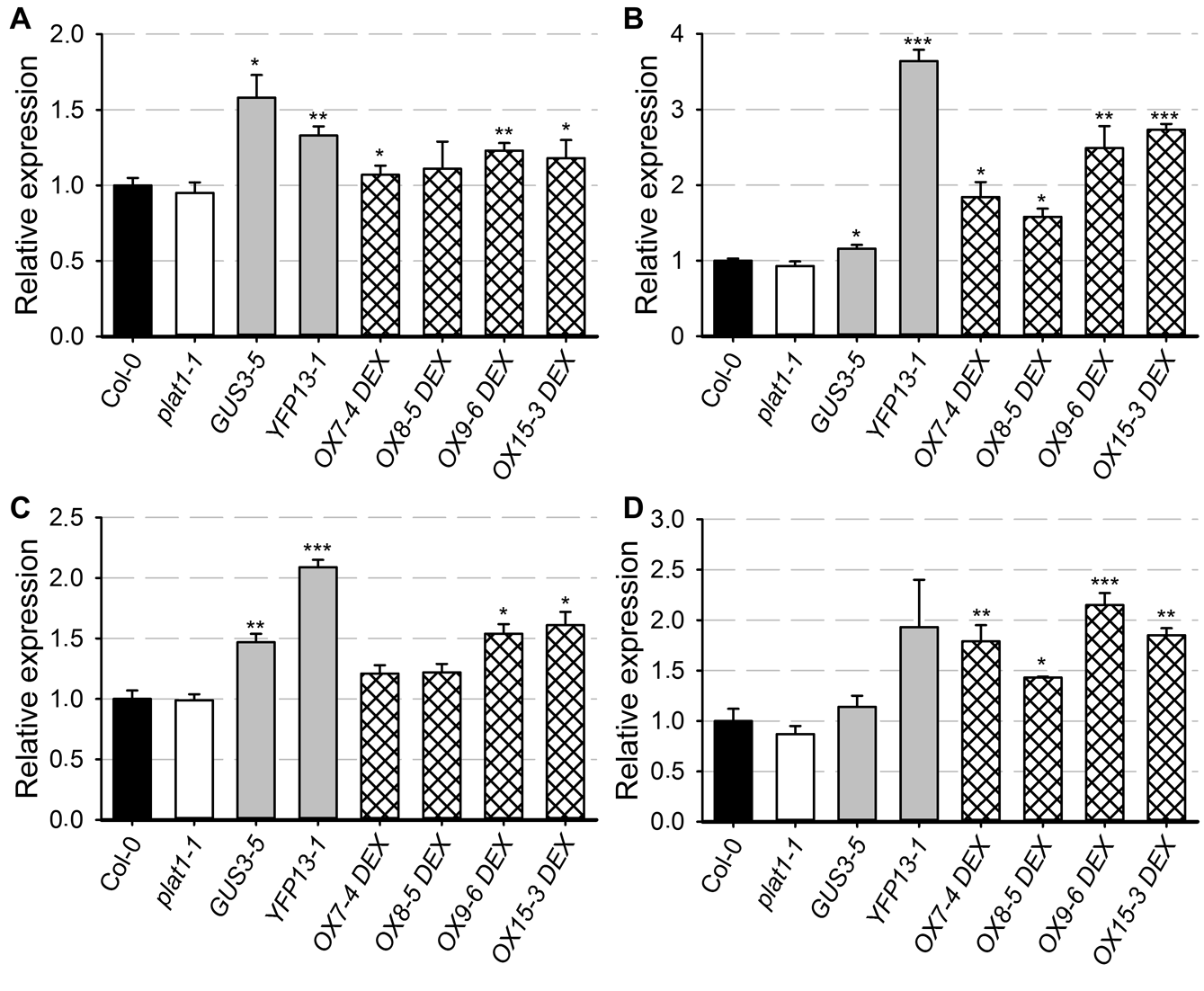

Supplement: Figure S7 — PLAT1 overexpression lines exhibit higher basal expression levels for ER stress markers. Relative expression levels for ER stress markers in the different PLAT1 overexpression lines compared to wild-type (Col-0) and the plat1-1 mutant. (A) BIP1,2 (HSP70), (B) CNX1 (CALNEXIN1), (C) CRT1 (CALRETICULIN1) and (D) PDIL (PROTEIN DISULFIDE ISOMERASE-like). Values are means of 3 replicates ± standard deviation. n≥10 per replicate. ***, ** or * indicate statistical significance calculated using the unpaired Student's t-test at p<0.001, p<0.01 or p<0.05, respectively. (TIF) [file pone.0112946.s007.tif]
