# Supplementary material for: The Arabidopsis PLAT Domain Protein1 Is Critically Involved in Abiotic Stress Tolerance
Source: PLoS One. 2014 Nov 14;9(11):e112946. doi: 10.1371/journal.pone.0112946 (PMC4232524; doi:10.1371/journal.pone.0112946)
Supplement: File S1 — Combined file containing supporting tables. Table S1: List of primers used for cloning, genotyping of T-DNA insertion mutants and EMSA controls. Table S2: List of primers used for RT-PCR. Table S3: ABA levels are not affected by PLAT1. Table S4: PLAT1 transcript levels in the different overexpression lines. Table S5: PLAT1 promotes lateral root formation. (DOC) [file pone.0112946.s008.doc]

**Table S1. List of primers used for cloning**, **genotyping of T-DNA insertion mutants and EMSA controls.**

| **Oligo name** | **Sequences** (**5'-3'**) | **Purpose** |
| --- | --- | --- |
| PLAT1-F probe | CCGTGTTTACCTAGTCCGATCT | Northern probe |
| PLAT1-R probe | GCTTGACCGGACAATTGTTCC |
| PLAT2-F probe | CATGTCTTCCGAGCCCAGTCT |
| PLAT2-R probe | CGAAACTGGACAGTTATTCCG |
| PLAT3-F probe | ATGAGCCTCCGTCTTTACGAC |
| PLAT3-R probe | CTCTCACTCATGGTCACTTCC |
| PLAT1-1-F | GACAAAACCATCAAAAATTTCG | Genotype |
| PLAT1-1-R | ACGACCCAAGAAAGCTTTTTC |
| SALK LB2 | GCGTGGACCGCTTGCTGCAACT |
| PLAT1-2-F | AAATCTGACAGCAAATATATAAGC |
| PLAT2-F | TTTGGGTGGTATTTCTGGACC |
| PLAT2-R | TATGATCGAATCTGTACCGGC |
| pROK2 LB1 | TGGTTCACGTAGTGGGCCATCG |
| PLAT1-F cDNA | ATGGCTCGTCGCGATGTTCT | PLAT1 cDNA |
| PLAT1-R cDNA | TTAAACGACCCAAGAAAGC |
| PLAT1-F Resc | TTGGAATTCAGGTGAGAATGGATACTTGAG | PLAT1 rescue/reporter |
| PLAT1-R Resc | ATCGGATCCACGACCCAAGAAAGCTTTTTC |
| ABF1-F OX | CTCGAGATGGGTACTCACATTGATATC | ABF overexpression |
| ABF1-R OX | GAGCTCTTACCACGGACCGGTAAGGGT |
| ABF2-F OX | CTCGAGATGGATGGTAGTATGAATTTG |
| ABF2-R OX | TCTAGATCACCAAGGTCCCGACTCTGT |
| ABF3-F OX | CTCGAGATGGGGTCTAGATTAAACTTC |
| ABF3-R OX | CCCGGGCTACCAGGGACCCGTCAATGT |
| ABF4-F OX | CTCGAGATGGGAACTCACATCAATTTC |
| ABF4-R OX | CCCGGGTCACCATGGTCCGGTTAATGT |
| pPLAT1-F | GGATCCCCATCTCAATTCTCACATCA | PLAT1 promoter for EMSA |
| pPLAT1-R | GTCGACTTTGTTTTTTTCCGGTGAACG |
| WIP-F1 | GGATCCCTCGAGATGGCTCGTCGCGATGTT | mutated PLAT1 promoter for EMSA |
| WIP-R1 | GTATACACAGTCTGGATCATCTTC |
| WIP-F2 | GTATACTGTCCGGTCAAGCTTAGG |
| WIP-R2 | TCTAGATTAAACGACCCAAGAAAG |
| ABRE-F | AATTCCGGACACGTGGCGTAAGCT | EMSA controls |
| ABRE-R | AGCTTACGCCACGTGTCCGGAATT |
| mABRE-F | AATTCCGGACCTACAGCCTAAGCT |
| mABRE-R | AGCTTAGGCTGTAGGTCCGGAATT |
| ABF1-F | GGATCCATGGGTACTCACATTGATATC | ABF protein purification |
| ABF1-R | CTGCAGTTACCACGGACCGGTAAGGGT |
| ABF2-F | GGATCCATGGATGGTAGTATGAATTTG |
| ABF2-R | GTCGACTCACCAAGGTCCCGACTCTGT |
| ABF3-F | GGATCCATGGGGTCTAGATTAAACTTC |
| ABF3-R | GTCGACCTACCAGGGACCCGTCAATGT |
| ABF4-F | GGATCCATGGGAACTCACATCAATTTC |
| ABF4-R | GTCGACTCACCATGGTCCGGTTAATGT |

**Table S2. List of primers used for RT-PCR.**

| **Oligo name** | **Sequences (5'-3')** | **number of cycles** | **annealing** |
| --- | --- | --- | --- |
| PLAT1-F | CGTGTTTACCTAGTCCGA | 26 cycles | 56°C |
| PLAT1-R | CTCAATCTCAAAATCCTGCGT |
| PLAT2-F | CATGTCTTCCGAGCCCAG | 26 cycles | 60°C |
| PLAT2-R | CTCAACATCAAAGCTTTGGTA |
| PLAT3-F | ATGAGCCTCCGTCTTTACGAC | 35 cycles | 60°C |
| PLAT3-R | CTCTCACTCATGGTCACTTCC |
| BIP1,2-F | TCACTTGGGAGGTGAGGACTTT | 28 cycles | 62°C |
| BIP1,2-R | CTCACATTCCCTTCGGAGCTTA |
| PDIL-F | CTCGTGAAGCTGAGGGTATTG | 28 cycles | 62°C |
| PDIL-R | TGTGCGAAATCTAACTCAGAG |
| CRT1-F | AGACCTTAGTCTTCCAATTCTC | 26 cycles | 62°C |
| CRT1-R | CCATTGTAAGTAAGGATAGCATG |
| CNX1-F | ATGAGACAACGGCAACTATTTTCC | 26 cycles | 63°C |
| CNX-1-R | CCATAATCCTCATGTCCTTCACT |
| ACT-F | GGTAACATTGTGCTCAGTGGTGG | 22 cycles | 65°C |
| ACT-R | AACGACCTTAATCTTCATGCTGC |
| UBQ-F | TTGAAGACGGCCGTACCCTC | 22 cycles | 65°C |
| UBQ-R | CGCTGAACCTTTCAAGATCCATCG |

Cycle number used for the expression analysis, at which the PCR amplification was still in the linear range. No product for *PLAT3* was obtained, even at 35 cycles, while positive signals were obtained from genomic DNA.

**Table S3. ABA levels are not affected by PLAT1.**

| line | ABA (ng/g FW) | significance |
| --- | --- | --- |
| Col-0 | 3.14 ± 0.48 |  |
| *plat1-1* | 2,88 ± 0.35 |  |
| *GUS3-5* | 2.73 ± 0.76 |  |
| *YFP13-1* | 2.42 ± 0.70 | * |

Values are means of 3 replicates ± standard deviation, n ≥ 10 per replicate. * indicates statistical significance calculated using the unpaired Student’s *t*-test at p<0.05.

**Table S4. *PLAT1* transcript levels in the overexpression lines.**

| line | *PLAT1* | significance |
| --- | --- | --- |
| Col-0 | 1.00 ± 0.03 |  |
| *plat1-1* | 0.08 ± 0.03 | *** |
| *plat2* | 1.01 ± 0.06 |  |
| *GUS1-3* | 1.15 ± 0.05 | * |
| *GUS2-1* | 1.17 ± 0.07 | * |
| *GUS3-5* | 1.20 ± 0.27 |  |
| *YFP5-1* | 1.20 ± 0.07 | * |
| *YFP13-1* | 1.32 ± 0.05 | * |
| Col-0 DEX | 1.02 ± 0.10 |  |
| *OX7-4* mock | 1.00 ± 0.08 |  |
| *OX7-4* DEX | 1.29 ± 0.32 |  |
| *OX8-5* mock | 0.99 ± 0.05 |  |
| *OX8-5* DEX | 1.41 ± 0.08 | ** |
| *OX9-6* mock | 1.04 ± 0.12 |  |
| *OX9-6* DEX | 1.91 ± 0.13 | ** |
| *OX15-3* mock | 1.01 ± 0.10 |  |
| *OX15-3* DEX | 1.58 ± 0.10 | ** |

Relative expression values with Col-0 expression set to 1.00. Values are means of 3 replicates ± standard deviation. n ≥ 10 per replicate. ***, ** or * indicate statistical significance calculated using the unpaired Student’s *t*-test at p<0.001, p<0.01 or p<0.05, respectively.

**Table S5. PLAT1 promotes lateral root formation.**

|  | lateral roots | |  | primary root | |  | root apical meristem | |
| --- | --- | --- | --- | --- | --- | --- | --- | --- |
| line | number | sign. |  | length (mm) | sign. |  | length (µM) | sign. |
| Col-0 | 24.0 ± 3.9 |  |  | 45.8 ± 5.9 |  |  | 325.6 ± 21.4 |  |
| *plat1-1* | 20.1 ± 3.2 | ** |  | 44.4 ± 7.2 |  |  | 310.2 ± 25.2 |  |
| *plat2* | 25.1 ± 3.8 |  |  | 49.1 ± 5.0 |  |  | 331.9 ± 20.0 |  |
| *GUS1-3* | 23.0 ± 3.5 |  |  | 43.1 ± 4.5 |  |  | 336.8 ± 22.9 |  |
| *GUS2-1* | 22.8 ± 3.8 |  |  | 44.3 ± 6.1 |  |  | 339.6 ± 19.5 |  |
| *GUS3-5* | 20.2 ± 3.6 |  |  | 46.5 ± 4.2 |  |  | 318.5 ± 20.1 |  |
| *YFP5-1* | 23.1 ± 4.0 |  |  | 43.3 ± 4.7 |  |  | 336.5 ± 22.8 |  |
| *YFP13-1* | 24.6 ± 1.7 |  |  | 44.7 ± 4.6 |  |  | 332.3 ± 20.1 |  |
| *OX7-4* mock | 26.4 ± 4.1 |  |  | 49.2 ± 6.3 |  |  | 332.4 ± 16.9 |  |
| *OX8-5* mock | 25.7 ± 4.4 |  |  | 46.3 ± 6.2 |  |  | 324.5 ± 16.3 |  |
| *OX9-6* mock | 26.0 ± 2.2 |  |  | 49.1 ± 6.6 |  |  | 327.7 ± 18.7 |  |
| *OX15-3* mock | 24.2 ± 3.1 |  |  | 43.5 ± 6.3 |  |  | 323.2 ± 21.8 |  |
| Col-0 DEX | 24.8 ± 3.1 |  |  | 47.0 ± 7.1 |  |  | 333.4 ± 25.3 |  |
| *OX7-4* DEX | 36.8 ± 2.5 | *** |  | 48.5 ± 5.6 |  |  | 337.8 ± 15.9 |  |
| *OX8-5* DEX | 33.6 ± 4.1 | *** |  | 44.7 ± 6.4 |  |  | 330.9 ± 22.4 |  |
| *OX9-6* DEX | 39.6 ± 5.2 | *** |  | 51.2 ± 7.6 |  |  | 338.1 ± 30.2 |  |
| *OX15-3* DEX | 28.5 ± 3.5 | ** |  | 50.9 ± 5.4 |  |  | 327.3 ± 26.2 |  |

Values are means of 2 replicates ± standard deviation. n = 20 per replicate. *** or ** indicate statistical significance calculated using the unpaired Student’s *t*-test at p<0.001, or p<0.01, respectively.
